# Recombinant production of the therapeutic peptide lunasin

**DOI:** 10.1186/1475-2859-11-28

**Published:** 2012-02-29

**Authors:** Stuart Kyle, Kier AR James, Michael J McPherson

**Affiliations:** 1 Astbury Centre for Structural Molecular Biology, Institute of Molecular and Cellular Biology, Faculty of Biological Sciences, University of Leeds, Leeds LS2 9JT, UK

**Keywords:** Lunasin, Autoinduction, *E. coli*, Cellulose binding domain, Chemopreventive peptide

## Abstract

**Background:**

Lunasin is a chemopreventive peptide produced in a number of plant species. It comprises a helical region with homology to a region of chromatin binding proteins, an Arg-Gly-Asp cell adhesion motif and eight aspartic acid residues. *In vitro *studies indicate that lunasin suppresses chemical and oncogene driven transformation of mammalian cells. We have explored efficient recombinant production of lunasin by exploiting the *Clostridium thermocellum *CipB cellulose binding domain (CBD) as a fusion partner protein.

**Results:**

We used a pET28 vector to express a CBD-lunasin fusion with a hexahistidine tag and Tobacco Etch Virus protease site, to allow protease-mediated release of native lunasin. Autoinduction in *E. coli *BL21 (DE3) Star cells achieved expression of 3.35 g/L of CBD-lunasin fusion protein. The final yield of lunasin was 210 mg/L corresponding to 32% of the theoretical yield. Purification by cellulose binding and nickel affinity chromatography were tested with the latter proving more satisfactory.

The effects of CBD-lunasin expression on growth and morphology of the *E. coli *cells were examined by light and electron microscopy revealing an altered morphology in a proportion of cells. Cell division appeared to be inhibited in these cells resulting in elongated, non-septated cells.

**Conclusions:**

The use of CBD as a fusion partner gave high protein yields by autoinduction, with lunasin release by TEV protease cleavage. With some optimisation this approach could provide a potentially valuable route for production of this therapeutic peptide. Over-expression in the host cells manifest as a cell division defect in a population of the cells, presumably mimicking some aspect of the chemopreventive function observed in mammalian cells.

## Background

**Figure 1:**
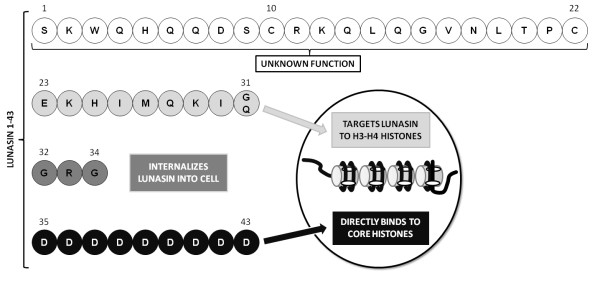
**Amino acid sequence of lunasin and activity of regions of the peptide**. Lunasin is a 43-amino acid peptide consisting of four regions. The function of residues 1-22 is currently unknown. Residues 23-31 target lunasin to chromatin. The Arg-Gly-Asp cell recognition sequence of residues 32-34 is known to internalise lunasin into the cell nucleus. The poly-aspartyl end of lunasin (residues 35-43) is responsible for binding it to the core histones within the chromatin. Adapted from [4].

Lunasin is a 43 amino acid chemopreventive peptide initially identified in soybean and more recently in barley, wheat, and *Solanum nigrum *[1]. It is a small subunit peptide derived from the larger cotyledon-specific 2S albumin (Gm2S-1) complex and comprises three distinct regions. An N-terminal helical region that shares homology with the conserved region of the chromatin binding proteins, an Arg-Gly-Asp (RGD) cell adhesion motif and a C-terminal sequence of eight aspartic acid (D) residues [2]. Various studies have shown the ability of lunasin to suppress both chemical and oncogene driven transformation of mammalian cells. The chemopreventive properties have also been illustrated in mouse skin cancer models where lunasin has been shown to suppress transformation and subsequent carcinogenesis [3]. The proposed biological activity of the regions of lunasin is shown in Figure 1.

There are two broad strategies for the production of therapeutic peptides and proteins: chemical synthesis and recombinant production by transgenic organisms ranging from bacteria and fungi to plants and animals. Chemical synthesis is rapid and effective for the production of custom-made peptides in relatively small quantities but can be costly and problematic during process scale-up and as amino acid sequence length increases with sequences over 35 amino acids not generally considered to be economically feasible [5]. In addition, the process employs chemicals that present potential environmental hazards. The use of microbial 'biofactories' for protein synthesis is widely employed in industry owing to their ease of use, robustness and costs. Recombinant systems are more effective for the production of long peptides (> 35 amino acids) and proteins.

The Gram-negative bacterium *E. coli *is one of the most attractive systems for heterologous protein production due to its ability to grow rapidly to a high cell density on inexpensive substrates and it is genetically well characterised. A common strategy for the production of recombinant peptides involves genetic attachment to a well expressed protein to generate a fusion protein. The protein acts to protect the peptide from proteolytic digestion and can protect the host from potential toxicity associated with some peptides. There are a number of fusion partner proteins available which differ in size and properties; some facilitate soluble expression while others promote the formation of inclusion bodies [6-8].

Previous reports of lunasin expression in *E. coli *have included its fusion to an 8 amino acid FLAG peptide epitope to allow antibody detection. It was observed that the DH5α host exhibited poor growth and an inability to septate, resulting in the formation of filamentous cells [9]. In another study, lunasin was expressed as a fusion to green fluorescent protein (GFP) using an *in vitro *transcription/translation reaction. While reasonable protein yields of 270 μg of purified GFP-lunasin protein were obtained from a 10 mL reaction, this only equates to 27 mg/L of fusion protein and a theoretical maximum of 4.1 mg of lunasin peptide. In addition the equipment and reagents required for this system are expensive which is likely to limit scale-up [10]. More recently lunasin has been expressed with a C-terminal His-tag in a soluble form and purified by immobilized metal affinity chromatography. Using an *E. coli *T7 system with IPTG induction, 4.73 mg of lunasin was expressed/L of culture [11]. This study showed that bacterial growth and expression levels were not affected by the presence of lunasin. In the present study, to enhance the levels of production of lunasin we have investigated the use of a His-tagged *Clostridium thermocellum *CipB cellulose binding domain (CBD) as a fusion protein and purification tag for the production of the native therapeutic peptide lunasin.

## Results

### Cloning of CBD-lunasin into pET28c

**Figure 2:**
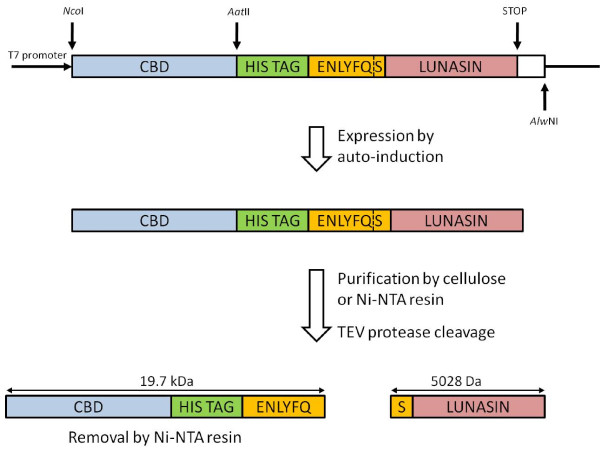
**Expression strategy of the CBD-lunasin fusion protein and subsequent TEV cleavage**. The arrangement of regions of the construct are shown. Lunasin peptide released following TEV cleavage has the native sequence as the N-terminal serine residue is provided by the TEV protease recognition sequence. Also indicated are the expected sizes of the resulting fragments.

The lunasin coding sequence with an N-terminal six histidine tag and TEV protease (TEVp) site was constructed by recursive PCR using the primers, EcHisTEVlunF and EcHisTEVlunR. This fragment was then cloned into pET28 downstream of the CBD coding region. DNA sequence analysis confirmed the integrity of the construct. Figure 2 shows a schematic representation of the recombinant protein expression and peptide release strategy.

### Expression of CBD-lunasin fusion protein from *E. coli *BL21* (DE3) by auto-induction

Auto-induction allows cultures to reach very high cell densities before recombinant protein expression occurs [12]. This approach reduces the risk of cell toxicity while enhancing product recovery.

**Figure 3:**
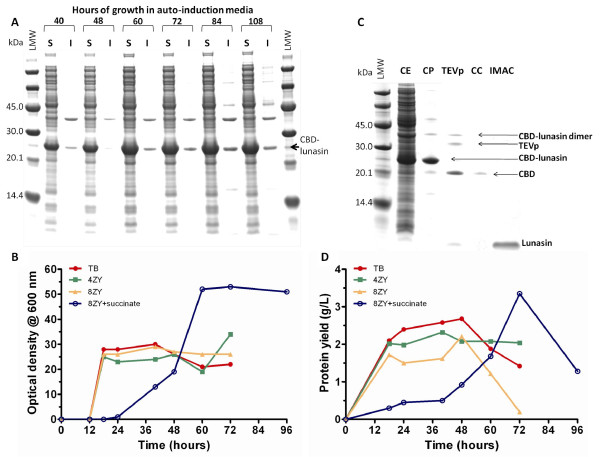
**Expression of CBD-lunasin constructs and protein recovery. **(A) SDS-PAGE analysis of soluble and insoluble fractions of E. coli BL21*(DE3) carrying pET28-CBD-lunasin grown on 8ZY auto-induction media supplemented with 4 × 5052 and succinate at 25°C. (B) Time-course of cell growth as OD600 in various auto-induction media at 25°C (C) SDS-PAGE analysis of the CBD-lunasin fusion protein purified using cellulose phosphate batch binding, cleavage by TEV protease and either a Microcon® concentrator column or Ni-NTA agarose step. (D) CBD-lunasin fusion protein yield over time-course in (B).

Initial expression trials with TB media were conducted over a period of 72 h at 25°C. This temperature has previously been shown to be optimal for the production of soluble CBD and CBD fusions within the laboratory (unpublished observations). It was apparent from SDS-PAGE analysis that the majority of the fusion protein was in the soluble fraction with yields reaching 1.4 g/L between 60 and 72 h of growth (Figure 3A).

From the work of Studier [12] and previous studies in our laboratory [13,14], it was clear that alternative basal media together with increased levels of carbon source could result in increased yields of fusion protein.

This was investigated with cultures grown over a period of 96 h at 25°C in four different media (TB, 4ZY, 8ZY and 8ZY). Samples were taken at regular intervals, and the culture optical densities at 600 nm (Figure 3B) and protein yields (Figure 3C) were determined. Maximal culture densities were obtained following 72 h growth at 25°C, in 8ZY medium supplemented with 4 × 5052 (2% glycerol, 0.2% glucose, 0.8% α-lactose monohydrate) and 25 mM succinate (Table 1) [12]. This culture reached very high culture densities with an OD_600 _value of 53. SDS-PAGE analysis in conjunction with quantification of yields by densitometry revealed that this medium resulted in the production of 3.35 g/L of CBD fusion protein at 72 h. Purification of the CBD-lunasin fusion protein resulted in a yield of 51.5% which equates to a maximum theoretical lunasin yield of 350 mg/L.

**Table 1:** Components of auto-induction media.

Medium components		Growth Medium		
	**TB**	**4ZY**	**8ZY**	**8ZY + succinate**

Tryptone (g/L) [Z]	12	40	80	80

Yeast extract (g/L) [Y]	24	20	40	40

NPCS (mM)				

Na_2_HPO_4_	25	25	25	25

KH_2_PO_4_	25	25	25	25

NH_4_CI	50	50	50	50

Na_2_SO_4_	5	5	5	5

MgSO_4_(mM)	2	2	2	2

Trace metals	0.2×	0.2×	0.2×	0.2×

5052 (%)				

Glycerol	2	2	2	2

Glucose	0.2	0.2	0.2	0.2

Lactose	0.8	0.8	0.8	0.8

Succinate (mM)	-	-	-	25

Adapted from [12]

### Purification of CBD-lunasin fusion protein by batch binding to cellulose

The low cost of cellulose makes this an attractive purification matrix and so we explored the recovery of the CBD-lunasin using cellulose. Following cell lysis the supernatant was diluted 2-fold with 200 mM Tris-HCl (pH 7.0) and prewetted cellulose phosphate was added to a final concentration of 200 mg/mL. After binding overnight at room temperature, the cellulose phosphate was washed twice with 100 mM Tris-HCl (pH 7.0) (Figure 3D). A number of elution steps typically 4, each with 25 mL, water were required to release around 50 mg of the protein thus resulting in a significant dilution of the fusion protein.

We therefore explored purification using nickel affinity chromatography (Ni-NTA) by exploiting the histidine tag. This allowed the more efficient recovery of concentrated fusion protein, with some evidence on SDS-PAGE of dimers and trimers of the fusion protein (Figure 3D).

TEV cleavage of the lunasin peptide from the CBD fusion protein results in release of lunasin with a native N-terminal serine provided by the TEV protease site ENLYFQ/S (Figure 2). TEV protease cleavage was conducted at room temperature over a 24 h time-period with a TEV protease concentration of 700 nM. Reasonably efficient cleavage was achieved although low levels of uncleaved fusion protein remained (Figure 3D). The dimeric form of the fusion protein appeared not to be susceptible to cleavage perhaps due to steric interactions between monomers occluding the protease sites.

Following TEV cleavage the lunasin peptide was separated from CBD, TEVp and uncut CBD-lunasin. Two approaches were investigated; the first was Microcon^® ^centrifugal filter devices that have a cellulose matrix acting as a molecular sieve to allow passage of molecules of less than 10 kDa, such as lunasin, whilst retaining larger molecules. The second approach was Ni-NTA agarose batch binding as all the proteins contain a His tag whilst lunasin does not and so would remain in solution (Figure 3D).

The Microcon^® ^centrifugal approach was ineffective as the majority of the peptide was retained. In contrast, batch binding with the Ni-NTA agarose proved to be quite efficient with 60% of the lunasin being recovered, corresponding to a yield of 210 mg/L culture.

To confirm the molecular mass and purity of the final lunasin product, mass spectrometry was performed and revealed a single major peak corresponding to 5024.09 Da in excellent agreement with the calculated mass of 5028.36 Da.

### Effect of lunasin on *E. coli cell morphology*

**Figure 4:**
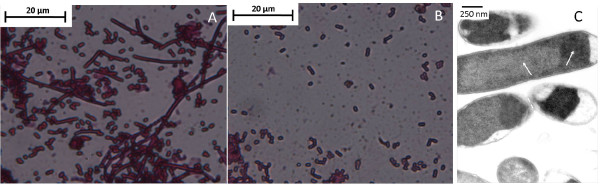
***E. coli* cell morphology expressing CBD-lunasin.** Light microscopy image of Gram stained E. coli BL21* (DE3) expressing (A) CBD-lunasin fusion protein and (B) CBD alone. Note the absence of the elongated non-septate forms of E. coli BL21*(DE3) in cells expressing CBD alone. (C) TEM image of the elongated, non-septate morphology of subsets of E. coli BL21*(DE3) expressing the CBD-lunasin fusion protein. Two distinct regions can be identified which are indicated by white arrows. It is likely that the darker regions represent the aggregation of fusion protein into insoluble inclusion bodies. In contrast the lighter regions are likely to represent the soluble fusion protein.

To check for any effect of the CBD-lunasin fusion on the growth and morphology of the *E*. *coli *BL21*(DE3) host cells, culture pellets were re-suspended in water and Gram stained (Figure 4A). By light microscopy it was apparent that expression of the CBD-lunasin fusion protein resulted in an altered morphology of some cells. Cell division appeared to be inhibited in a proportion of the cells resulting in elongated, non-septated cells with lengths of over 100 μM. In comparison, a Gram stain of *E. coli *BL21* (DE3) expressing CBD alone did not show such elongated, non-septate cells (Figure 4B).

To further investigate the morphology of these elongated *E. coli *cells, samples were visualized by transmission electron microscopy (TEM) (Figure 4C). Many of the cells appeared able to successfully undergo cell division as the invaginations of the cell wall, which precede cell division, were observed. In contrast no such invaginations of the cell wall were present in the elongated *E. coli *cells indicating that cell division had been inhibited. Lunasin does not appear to elicit an inhibitory effect on cell division phenotype in all host cells.

## Discussion

In this work, the *C. thermocellum *derived CBD protein was used as a fusion partner for the expression of the therapeutic peptide lunasin. The CBD protein appears to reduce the previously observed toxicity of the lunasin peptide [15], as extremely high cell culture densities were obtained (OD_600 _of 53) with correspondingly high recovery of fusion protein. From the growth curves the highest culture densities and yields were obtained from cultures grown in very rich 8ZY medium supplemented with 4 × 5052 and succinate. However, use of this medium does result in a prolonged initial growth lag phase and so simpler TB medium may prove more suitable for large scale applications. Although the culture densities are lower for this medium it more rapidly reaches its maximal culture density. The culture time saved together with the lower cost of TB medium may compensate for the reduced peptide yields.

The use of the CBD fusion protein potentially offers significant advantages for the production of lunasin and other peptides. The ease of the batch purification procedure and the high degree of purity of the final product in conjunction with a cheap and abundant cellulose purification matrix are highly desirable for scale-up production. One challenge with this approach is determining efficient elution conditions that do not result in large volumes of dilute protein. In this regard the nickel affinity purification was more appropriate with around 50% recovery of fusion protein. Improvements in peptide recovery might be achieved by combining cellulose affinity with on column cleavage followed by immobilised metal affinity chromatography (IMAC) to remove contaminants. As recovery of peptide was only 60% from the IMAC step, some of the target peptide may bind non-specifically to the IMAC resin resulting in peptide loss and so separation by alternative chromatographic approaches following TEV cleavage could be explored. A final Detoxi gel™ column would allow the removal of endotoxins resulting in a highly purified peptide solution that could then be used for cell-based assays and ultimately *in vivo *models.

From visualisation of *E. coli *cells expressing the CBD-lunasin, it was apparent that the lunasin peptide can exert a significant effect on cell morphology, but this was only apparent in a subpopulation of cells. A similar phenotype has been observed in *E. coli *strains carrying mutations in the filamentous temperature sensitive (Fts) Z gene [16]. The FtsZ gene shares homology with the tubulins of eukaryotic cells and has led to the proposal that lunasin may have a similar effect on eukaryotic cells as FtsZ mutations in bacteria [9,16]. Lunasin expression in *E. coli *has a similar cytokinesis inhibitory effect that can be prevented by the removal of the polyaspartyl tail of lunasin, indicating that aberrant cell division is attributable to this acidic domain [9]. The elongated phenotype could be due to the direct interaction of lunasin with one or more of the proteins or may be indirect due to interactions with other protein(s) within *E. coli *perhaps preventing *FtsZ *gene transcription. Clearly the effect on cell division is variable as this was not observed in a recent study [11]. Our observations reveal that only a sub-population show the elongated morphology. This could be due to a dosage effect perhaps reporting the release of lunasin from the fusion protein, such that in a subpopulation of cells higher levels of lunasin are released and reach a threshold level able to mediate the observed phenotype. Further investigation of the difference observed between the various lunasin expression studies with regard to aberrant cell division would be required to understand this phenomenon.

The altered cell morphology may explain the very high culture densities observed as the elongated cells may alter the relationship between OD_600 _and cell number. The next stage will be to compare the efficacy of the recombinantly produced peptide with that from native sources such as soybean. For example through *in vitro *trials on cancer cell lines such as human breast cancer MCF-7 cells to determine whether cell division is inhibited and subsequently *in vivo *testing perhaps utilising skin cancer mouse models.

## Conclusions

The CBD protein has been used as a successful fusion partner for the production of the chemopreventive peptide lunasin resulting in yields of peptide of upto 210 mg/L. Optical densities and protein yields were found to be enhanced when basal media was supplemented with 4 × 5052 and with succinate. However, these yields could potentially be increased further in bioreactors where oxygen and nutrient levels can be tightly regulated. This study highlights the potential for producing a therapeutic peptide in a prokaryotic system and may be of general applicability for a range of other therapeutic peptides.

## Methods

### Bacterial strains, plasmids and cell culture media

*E. coli *XL1 Blue (Stratagene, La Jolla, CA) was used for routine cloning. *E. coli *BL21Star (DE3) (Invitrogen, Carlsbad, CA) was used as the expression strain. The expression vector pET28c was from Novagen, Madison, WI and pGEM-T Easy from Promega.

The auto-induction growth media Terrific Broth (TB), 4ZY, 8ZY, 8ZY supplemented with succinate were prepared according to [12]. The composition of various auto-induction media are given in Table 1. Kanamycin and carbenicillin were added to media to a final concentration of 50 μg/mL as appropriate Cultures were grown in an orbital incubator at 37°C with shaking at 220 rpm unless otherwise stated.

### Construction of plasmid pET28c/CBD/his/TEV

We used a plasmid generated in the laboratory by Dr. J. Riley. pET28c/rub/His/TEV was restricted with *Nco*I and *Aat*IIand treated with antarctic phosphatase (New England BioLabs, Hitchin, UK) to generate the plasmid backbone. A pUC57 plasmid containing the *C*. *thermocellum *CBD coding sequence codon optimised for expression in *E. coli *(GenScript, Piscataway, NJ) was restricted with the same enzymes and the CBD insert was gel extracted, purified using a QIAquick Gel Extraction Kit (Qiagen, Crawley, UK) and ligated into the vector.

Following transformation into *E. coli *XL-1 blue cells, kanamycin resistant colonies were screened by colony PCR using T7 forward (TAATACGACTCAACTATAGGG), T7 reverse (GCTAGTTATTGCTCAGCGG) primers. Clones generating a 814 bp band were then confirmed by DNA sequencing on an ABI 3100XL Capillary Sequencer (DNA Sequencing Facility, Biological Sciences, University of Leeds).

### Gene synthesis by recursive PCR

In order to generate the lunasin coding sequence, recursive PCR was performed with two primers, EcHisTEVlunF (GCGACGTCCATCACCATCACCATCACGAAAACCTGTATTTTCAGAGCAAATGGC AGCATCAGCAAGATAGCTGCCGCAAACAGCTGCAGGGCGTGAATCTGAC) and EcHisTEVlunR (GCACAGTGACTGTTATTAATCATCGTCATCATCATCGTCATCATCGCCGCGGCCC TGAATCTTTTCCATGATATGTTTTTCGCACGGGGTCAGATTCACGC) possessing 13 bp complementary regions at their 3' ends (underscored). Primers were added to a final concentration of 0.3 μM to a PCR reaction mixture consisting of 1 × KOD Hot Start DNA polymerase buffer (20 mM Tris-HCl (pH 7.5 at 25°C), 8 mM MgCl2, 0.5 mM DTT, 50 mg/mL BSA) (Novagen), 0.2 mM each dNTP, 1 mM MgSO4 and 1 U KOD Hotstart DNA polymerase and made up to 50 μL with sterile ddH2O. PCR was performed in a G-storm GS2 PCR machine (Gene Technologies) at 95°C for 5 min then 39-45°C for 30 s and 72°C for 1 min.

The PCR product was cloned into pGEM T easy according to the manufacturer' guidelines (Promega) transformed into *E*. *coli *XL-1 blue cells. Colonies containing recombinant plasmids were identified by colony PCR, as above, with the primers T7 Forward and SP6 promoter (ATTTAGGTGACAGTATAGAATAC). The insert was recovered by *Aat*II and *Alw*NI restriction digestion ligation into similarly digested pET28c/CBD/His/TEVp vector. Following colony PCR screening the sequence of pET28c/CBD/His/TEVp/lunasin was confirmed by DNA sequencing.

### *E. coli *expression of pET28c constructs by auto-induction

The protocols described by [12] were followed. LB plates with kanamycin (50 μg/mL) were streaked from glycerol stocks and grown overnight. A single colony was used to inoculate 2 mL of LB medium with kanamycin (50 μg/mL and grown at 37°C, 200 rpm for 6 h. A 200 μL aliquot of this starter culture was then used to inoculate 400 mL of TB, 4ZY, 8ZY or 8ZY supplemented with 25 mM succinate and auto-induction media components (Table 1) in baffled flasks. Cultures were grown at 25°C in an orbital incubator at 250 rpm. Samples of 1 mL were taken at regular intervals and the cell pellets stored at -20°C for time course expression analysis by SDS-PAGE. After 60-96 h the cultures were harvested by centrifugation, 5000 × *g *for 20 min at 4°C and the cell pellets stored at -80°C prior to processing.

### *E. coli *expression of TEV protease and protein concentration analysis

Expression of TEV protease was performed according to the method devised by [17]. Initially two pre-cultures of 5 mL LB containing ampicillin (100 μg/mL) and chloramphenicol (34 μg/mL) were grown over night at 37°C and used to inoculate two 500 mL volumes of LB in 2 L flasks. These cultures were grown to an OD_600 _of 0.6 and IPTG was added to a final concentration of 1 mM and the temperature reduced to 20°C for 20 h. The cells were then harvested by centrifugation at 5000 × *g *for 15 min (4°C) and re-suspended in 40 mL of lysis buffer (50 mM HEPES, 25% w/v sucrose, 5 mM MgCl2, 1% v/v Triton^® ^X-100 at pH 8.0 supplemented with 200 units/mL of Omnicleave nuclease (Epicentre, Cambridge, UK) and 1 mg/mL of lysozyme, prior to use). After cell lysis for 3 h on a 'Roller Mixer SRT1' (Stuart Scientific) at 4°C cell debris was removed by centrifugation at 15,000 × *g *for 30 min. The supernatant was dialysed into 5 L of dH_2_0 containing 50 mM NaH_2_PO_4_, 300 mM NaCl, pH 7.0 at 4°C overnight and subsequently purified by nickel affinity chromatography. Following purification, EDTA and DTT were added to a concentration of 2 and 10 mM respectively. The eluate was then dialysed against 5 L of dH_2_0 containing 25 mM NaH_2_PO_4_, 200 mM NaCl, 2 mM EDTA and 2 mM DTT (pH 7.0) over night at 4°C. Samples were concentrated to 30 μM using an 'Amicon Ultra^®^' centrifugal device with a molecular cut off point of 3500 Da.

The protein concentration was determined spectrophotometrically and calculated using the extinction coefficient ε_280 _= 36130 M^-1 ^cm^-1 ^[17] in conjunction with the Beer-Lambert law (A = εcl).

### Analysis of soluble and insoluble cell proteins

Cells were harvested at 5000 × *g *for 15 min (4°C) in a fixed angle rotor and lysed in lysis buffer as above using 5 ml/g of cell pellet for 3 h at 4°C. After centrifugation at 15,000 × *g *for 30 min the supernatant represented the soluble fraction. The pellets representing the insoluble fraction was resuspended in the original volume of lysis buffer supplemented with 8 M urea at 4°C with agitation for 2 h to solubilise insoluble fusion protein. Equal volumes of soluble and insoluble fractions were analysed by SDS-PAGE.

### SDS-PAGE analysis of protein expression and densitometry analysis

Protein samples were heated at 95°C for 10 min in loading buffer consisting of 40% SDS, 20% β-mercaptoethanol, 20% glycerol, and 0.01% of bromophenol blue in 1 M Tris-HCl (pH 6.8) before analysis on NuPAGE^® ^12% Bis-Tris gels (Invitrogen, Paisley, UK) at 200 V for 45 min. Protein bands were visualised using SimplyBlue™ SafeStain (Invitrogen, Paisley, UK) and protein concentrations estimated by densitometry using the AlphaImager Spot Denso software. The intensities of the bands were compared to those of known concentrations of low molecular weight protein standards (GE healthcare).

### Protein purification

#### Batch binding to cellulose phosphate

Cellulose phosphate was added (200 mg/mL) directly to soluble cell extract samples and incubated at room temperature with agitation for 2 h to allow binding of CBD to the cellulose. The cellulose was then washed twice with 10 mM sodium phosphate buffer (pH 8.0), with gentle agitation for 20 min followed by centrifugation at 7000 × *g *for 10 min at 4°C and the wash buffer was decanted. Finally, the bound protein was eluted by addition of cold ddH_2_0 and incubation for 1 h at 4°C. This step was repeated up to three times to improve protein recovery. Resulting purified fractions were analysed by SDS-PAGE. Where protein solutions were too dilute 'Microcon^®^' and 'Amicon Ultra^®^' centrifugal devices was used according to the manufacturers' guidelines, for volumes below and above 1 mL respectively, to concentrate the samples.

#### Nickel affinity chromatography (Ni-NTA)

Purification of His-tagged polypeptides and proteins was achieved by using a 5 mL HiTrap™ column containing Chelating Sepharose™ High Performance (GE Healthcare). Protein samples were resuspended in binding buffer (20 mM Tris-HCl, 0.5 M NaCl, 5 mM imidazole, 1 mM β-mercaptoethanol, pH 8) and loaded onto the column followed by extensive washing with binding buffer containing 20 mM imidazole to remove non-specifically bound proteins. The bound proteins were eluted by applying a 0-100% gradient (20 mM - 500 mM) over 50 mL of buffer and 1 mL fractions were collected. Fractions containing protein were analysed by SDS-PAGE analysis. Protein concentrations were determined by using the Bradford assay reagent (Sigma-Aldrich).

### TEV cleavage of peptide constructs

Cleavage experiments were performed according to [17]. TEV protease was used at a final concentration of 700 nM in TEV protease cleavage buffer (25 mM NaPi, 125 mM NaCl, and 5 mM DTT, pH 7.4) at room temperature.

### Mass spectrometry

Peptide samples were first concentrated using C18 ZipTip (Millipore UK, Ltd.) and eluted in 50% methanol/0.1% aqueous formic acid and then analysed by positive ionisation nanoelectrospray using an LCT Premier mass spectrometer (Waters Corporation, Manchester, UK). Samples were infused using a NanoMate (Advion Biosciences Inc., Ithaca, NY) autosampler and ionisation interface which was maintained at 4°C. A capillary voltage of 1.8 kV was set with a nitrogen gas flow of 0.4 psi for sample introduction and ionisation. The sampling cone voltage was optimised at 20 V, Ion Guide 1 was at 130 V and Aperture 1 was at 60 V. Data were acquired over the m/z range 500-5000, and data processing was performed using the MassLynx software supplied with the mass spectrometer. An external calibration using sodium iodide clusters was applied to the data.

### Gram-staining and light microscopy

Cultures (10 μl) containing *E. coli *BL21* (DE3) was smeared onto a slide and heat fixed. Crystal violet solution was applied and allowed to stand for 1 min before extensive washing. The slide was then flooded with iodine solution and allowed to stand for 1 min before rinsing again. The slide was then decolourised with acetone for 5 s and immediately rinsed with distilled water. Safranin was applied and allowed to stand for 30 s before a final rinse with distilled water. Bacterial slides were examined using a light microscope (Olympus, BX51) under a 100 × objective.

### Transmission electron microscopy

*E. coli *cells in growth media were harvested by centrifugation at 17,900 × *g *for 2 min in microcentrifuge tubes. Cells were subsequently washed twice with dH_2_O then fixed with 2.5% glutaraldehyde for 3 h followed by washing twice with 0.1 M phosphate buffer (pH 7.0) for 30 min each followed by centrifugation at 6,000 × *g *for 5 min to collect the cells. Cell samples were incubated in 1.0% osmium tetraoxide solution overnight followed by washing twice with 0.1 M phosphate buffer and harvested by centrifugation at 6,000 × *g *for 5 min.

Samples were then dehydrated by incubating in ascending concentrations of ethanol (20%, 40%, 60%, 80%, and 100% twice) for 30 min each. Dehydration of the samples was followed by two incubations in propylene oxide for 30 min followed by centrifugation at 6,000 × *g *for 5 min to harvest the cells. Embedding was achieved by incubating in a 50%:50% mix of propylene oxide and araldite overnight. Samples were then incubated in a 75%: 25% araldite:propylene mix for 3 h. Finally, samples were re-suspended in fresh araldite and transferred to embedding moulds and incubated overnight at 60°C to set. The embedded cells were sectioned using a microtome and placed onto Glow-discharged carbon coated copper TEM grids for visualization using a Philips CM10 TEM operating at 80 kV.

## Abbreviations

CBD: Cellulose Binding Domain; *FtsZ *gene: Filamentous temperature sensitive Z gene; IMAC: Immobilised Metal Affinity chromatography; Ni-NTA: Nickel Nitrilotriacetic Acid; TEV(p): Tobacco Etch Virus (protease); 5052: 0.5% glycerol: 0.05% glucose: 0.2% α-lactose monohydrate.

## Competing interests

The authors declare that they have no competing interests.

## Authors' contributions

SK assisted with microscopy analysis and wrote the paper. KARJ performed experimental work. MJM devised and co-ordinated the study and edited the paper. All authors read and approved the final manuscript.
